# Nano-Fibrous Networks from Co-Assembly of Amphiphilic Peptide and Polyelectrolyte

**DOI:** 10.3390/polym13223983

**Published:** 2021-11-18

**Authors:** Thomas Babut, Mona Semsarilar, Marc Rolland, Damien Quemener

**Affiliations:** Institut Européen des Membranes, IEM, UMR 5635, Univ Montpellier, ENSCM, CNRS, F-34090 Montpellier, France; thomas.babut8@gmail.com (T.B.); mona.semsarilar@umontpellier.fr (M.S.)

**Keywords:** membrane, peptide amphiphile, polyelectrolyte, self-assembly

## Abstract

Organize the matter on an increasingly small scale is sought in order to increase the performance of materials. In the case of porous materials, such as filtration membranes, a compromise must be found between the selectivity provided by this nanostructuring and a permeability in particular linked to the existing pore volume. In this work, we propose an innovative waterborne approach consisting in co-assembling peptide amphiphiles (PA) which will provide nanostructuring and polyelectrolytes which will provide them with sufficient mechanical properties to sustain water pressure. C_16_-V_3_A_3_K_3_G-NH_2_ PA nanocylinders were synthesized and co-assembled with poly(sodium 4-styrenesulfonate) (PSSNa) into porous nano-fibrous network via electrostatic interactions. The ratio between C_16_-V_3_A_3_K_3_G-NH_2_ and PSSNa was studied to optimize the material structure. Since spontaneous gelation between the two precursors does not allow the material to be shaped, various production methods have been studied, in particular via tape casting and spray-coating. Whereas self-supported membranes were mechanically weak, co-assemblies supported onto commercial ultrafiltration membranes could sustain water pressure up to 3 bars while a moderate permeability was measured confirming the existence of a percolated network. The produced membrane material falls into the ultrafiltration range with a pore radius of about 7.6 nm.

## 1. Introduction

Electrostatic interactions are known to provide a strong driving force towards stable co-assemblies of molecules, polyelectrolytes, or particles. If the presence of electrostatic charges ensures the solubility or the dispersibility of the objects, then charge compensation will often lead to an anarchical precipitation of the ensembles. Therefore, an additional strategy is required to build a material in order to control and predict its structural organization. For example, in the layer-by-layer technique (LbL), a polyelectrolyte is first coated onto a substrate, the excess is rinsed off and then a second complimentary charged polyelectrolyte is coated to grow the film [1,2,3]. In other cases, interesting complexes were scaled up forming nanomaterials made from oppositely charge precursors through charge compensation after mixing or diffusion of their aqueous solutions [4,5,6]. A particularly suitable molecule is peptide amphiphiles (PA) since they can self-assemble in a robust and predictable way. They can form gels and can have a charged sequence in its structure. PA are a family of self-assembling molecules consisting of a hydrophobic alkyl chain attached to a peptide sequence [7,8]. Interestingly, if a β-sheet forming peptide sequence is inserted, then the assembly is driven towards mono dimensional nanostructures such as cylindrical nanofibers [9]. A peptide sequence carrying charges could be incorporated as well in order to improve the amphiphilicity and possibly to bring bioactivity in the frame of biomedical applications [10].

The presence of charges has been used to trigger a higher level of assembly and obtain for example nanofibrous materials through controlled charge neutralization or through electrostatic interactions. As an example, Bulut et al. have reported Lauryl-VVAGK PA-oligonucleotide (ODN) hydrogel thanks to a charge screening for the cellular delivery of ODN in the scope of gene therapy [11]. In another work, a complex between heparin and PA has been studied by Rajangam et al. as a binding gel substrate for signaling molecules such as angiogenic growth factor. Here, it is the rigidity of an amphiphilic peptide network to be compared to that of a hydrogel formed from a polymer which was sought so as to better orient the growth factors and thus maximize their activity [12]. The combination of two oppositely charged PAs was found to self-assemble into nanofibers, enabling the bringing of different bioactive epitopes into a gel network [13]. The same strategy was later used in conjunction of building an independent poly(ethylene glycol) hydrogel as a versatile synthetic mimics of natural extracellular matrix for tissue engineering and regenerative medicine applications [14]. A very large contribution in this field can be attributed to the team of S. Stupp, with in particular the preparation of nanofibrous networks formed from a high molecular weight polyanion (hyaluronic acid, HA) and a positively charged PA C_16_V_3_A_3_K_3_ [15]. Each component in a separated aqueous solution was put into contact and a well-ordered solid assembly was formed in milliseconds at the interface to yield hierarchical membranes with tunable water permeability [16]. The intrinsic structure of these membranes depends directly on the nature of the interactions between HA and PA [17] and can be altered on demand by an electric field [18]. Other polysaccharides were more recently tested such as alginate which self-assemble with Pro-K-(Phe-K)_5_-Pro into nanofiber network [19]. A LbL technique between alginate and lauryl-V_2_AGK_3_-Am PA has amplified the preparation scale towards multilayered biomaterials by repeating the alternate deposition of both components [20]. Self-assembling peptides–alginate composite hydrogel has demonstrated improved mechanical properties to serve as injectable biomaterial for bone regeneration [21]. In a comprehensive study, various polyelectrolytes such as λ-carrageenan or poly(acrylic acid) were studied together with heparin, alginate or hyaluronic acid in order to vary the aggregation strength with the PA, and the resulting membrane structure was discussed accordingly [22]. Bulk gels with lamellar microstructure were formed by vortexing solutions of chitosan and C_16_V_3_A_3_E_3_–OH with potential applications for protein delivery in regenerative medicine and wound healing [23]. Finally, poly(sodium 4-styrenesulfonate) (PSSNa) hydrogels were reported by Radvar et al. with sulfonate groups chosen to promote hydrogel mineralization, as well as binding sites for basic proteins. This dual functionality enables to retain and control the release of charged proteins with potential application in 3D environments for cell differentiation [24].

In the race to the production of high performance materials, controlling their structure/morphology at the lowest scale has become vital. The nanofabrication process becomes more complex when a percolating porosity is required in applications, such as in filtration membranes. In that case, one strategy to introduce a porous volume is to use nano-building blocks which will leave a space between them after assembly [25,26,27,28,29,30,31,32,33]. Here, the outstanding properties of PA molecules to self-assemble into nanofibers are combined to the mechanical properties of polyelectrolytes to yield porous composite materials. The objective of this work is to design new membrane materials via the mixture of a charged PA and a polyelectrolyte. These two precursors being water-soluble, a waterborne membrane production strategy is developed which fits with the objective of minimizing the environmental impact of membrane preparation. In this work, C_16_-V_3_A_3_K_3_G-NH_2_ PA was synthesized and co-assembled in water with PSSNa into porous nanofibrous network. As stated above, mixtures of PA and polyelectrolytes generally produce spontaneous gels, which makes difficult to shape the membrane. Thereby, the ratio between PA and the polyelectrolytes was studied to optimize the material structure. Porous films were elaborated by tape casting and spray coating. Whereas self-supported membranes were mechanically weak, co-assemblies supported onto commercial ultrafiltration membranes could sustain water pressure up to 3 bars while a moderate permeability was measured confirming the existence of a percolated network. The produced membrane material falls into the ultrafiltration range with a pore radius of about 7.6 nm.

## 2. Materials and Methods

### 2.1. Materials

Rink amide AM-PS crosslinked 1%; DVB 100–200 mesh 0.87 mmol/g (SENN CHEMICALS); Glycine: Fmoc-Gly-OH ≥ 98% (ALDRICH); Lysine: Fmoc-Lys(Boc)-OH ≥ 98% (ALDRICH); Alanine: Fmoc-Ala-OH 95% (ALDRICH); Valine: Fmoc-Val-OH ≥ 98% (ALDRICH); BOP: benzotriazol-1-yloxytris(dimethylamino)phosphonium hexafluorophosphate ≥ 99.0% (ALDRICH); DIC: Diisopropylcarbodiimide 99% (ALDRICH); HOBt: Hydroxybenzotriazole ≥ 97% (ALDRICH); Palm-Cl: Palmitoyl Chloride ≥ 98% (ALDRICH); TFA: Trifluoroacetic acid ≥ 99% (ALDRICH); PSSNa: poly(sodium 4-styrenesulfonate) Mw = 70,000 g/mol (ALDRICH); Fmoc Rink amide resin Piperidine; 1,8-diazabicyclo[5.4.0]undéc-7-ène (DBU); 1-Hydroxybenzotriazole (HOBt); N,N′-Diisopropylcarbodiimide (DIC); Dimethylformamide (DMF); dichloromethane; methanol; diethylether were purchased from Merck.

### 2.2. Methods

PA synthesis. Fmoc Rink amide resin (1.15 g, 0.87 mmol/g loading, 1 mmol) was used after removing of the fmoc protecting group using a mixture of piperidine/DBU/DMF (2:2:96) following by several rinsing steps with dichloromethane and methanol. In a SPPS glass reactor charged with deprotected resin was added 1-Hydroxybenzotriazole (HOBt) (0.41 g, 3 mmole), N,N′-Diisopropylcarbodiimide (DIC) (0.5 mL, 3 mmole) and 3 mmol of the Fmoc Amino acid in DMF. The coupling reaction was carried out for 3 h followed by several rinsing steps with dichloromethane and methanol. At the end of each coupling step, a Kaiser test was performed to ensure the completion of the reaction. After the removal of the Fmoc protecting group, this coupling step was repeated to obtain H-Val-Val-Val-Ala-Ala-Ala-Lys-Lys-Lys-Gly-resin sequence. A last acylation step was carried out on the peptide-resin with a solution of palmitoyl chloride in dichlorometane (10% *v*/*v*, 8 mL) for 1 h. The resin bound protected peptide chain was then treated with a mixture (6 mL) of trifluoroacetic acid (TFA) containing 5% (*v*/*v*) of distilled water. After 5 h the resin was filtered off and the solution was precipitated in diethylether. The resulting PA (Mw = 1207.66 g/mol) was collected, dried, resolubilized in distilled water and lyophilized (yield 90%). After characterization by MS (MH^+^ = 1207.93 g/mol), PA was used without further purification step.

Membrane preparation. Tape-casting: 300 μL of solution are deposited with a knife 250 μm high. The speed of movement of the knife is 1 cm/s. The solutions are applied using a syringe to form a strip of 3 mm wide and 18 mm long. This solution is spread either directly on a glass slide or on a hydrophilic PVDF filter 25 mm in diameter (Durapore Membrane, hydrophilic PVDF, 0.45 µm, 25 mm, Millipore). The tape-casting was performed on an Elcometer 4350.

Spray-coating. The PSSNa solution injected is 6 g/L. The deposition pattern is a rectangle 30 mm by 75 mm. This rectangle is divided into three sections in the width (30 mm). The nozzle passes through each section. The nozzle is located 60 mm from the sample, the speed 250 mm/min and the flow is set to 100 mL/h. The spray-coating was carried out on a ND-SP from Nadetech.

Scanning electron microscopy. SEM analyses were performed using a Hitachi S-4500 instrument operating at a spatial resolution of 1.50 nm at an energy of 15 kV. The samples were dried and covered with an ultra thin layer of electrically conductive Platinum deposited by high vacuum evaporation.

Rheology. The rheology analyzes were carried out on a Physica MCR 301 from Anton Paar with a conical spindle: CP50-1. The mixtures are carried out directly on the rheometer stage to minimize changes in the medium.

Water permeation. The filtration studies were performed in a 10 mL filtration cell with 25 mm diameter. The fluid is pressurized through the membrane using compressed air. The pressure drops studied are between 0 and 3 bar. For the filtration tests, the prepared membrane (d = 2.5 cm) was placed in a 10 mL filtration cell (Amicon 8010 stirred cell). Then, the filtration cell was connected to a water tank and a compressed air line. The mass of water passing through the membrane (permeate) was recorded with SartoConnect software during 20 min for each pressure drop. All filtration experiments were performed at 25 °C with dust free ultrapure water (filtered through a 400 μm filter).

AFM. AFM images were taken with tips oscillating at a frequency of 300 kHz in intermittent contact mode. The scan speed is 0.74 lines per second. Parameters such as gain, setpoint or drive are determined for each image and no value is preset. However, a low setpoint and drive are favored, as well as a gain close to 30% for the integral and proportional values. The approach percentage is set at 90%. AFM analyzes were performed on a PicoSPM II.

Circular dichroism (CD) spectroscopy. Circular dichroism solutions were performed in mQ water. The peptides (4 mg) were dissolved in 1 mL of water. The cuvette used for this analysis has an optical path of 0.2 mm. Circular dichroism analyzes were carried out on the following devices: PMS450; MOS450; ALX250; MM450 from ScienceInstrument.

## 3. Results and Discussion

**Figure 1:**
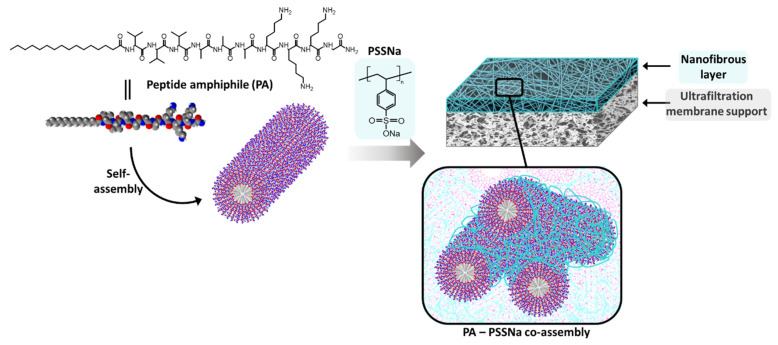
Schematic representation of a nanofibrous membrane from peptide amphiphile C_16_V_3_A_3_K_3_G-NH_2_ and poly(sodium 4-styrenesulfonate) (PSSNa) co-assembly.

C_16_-V_3_A_3_K_3_G-NH_2_ lipidated PA (Figure 1) has been synthesized using standard 9-fluorenylmethoxycarbonyl-based solid phase procedure. It integrates three valines and three alanines as structuring part, which impart a polarity gradient between the hydrophobic part C_16_ and the hydrophilic part K_3_G-NH_2_, known to help alignment of PA thus leading to robust cylindrical micelles containing β-sheet secondary structure in the V_3_A_3_ peptide domain [9].

**Figure 2:**
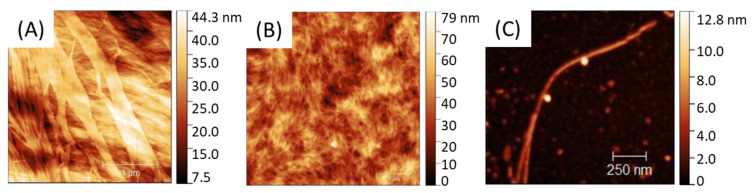
AFM of C_16_V_3_A_3_K_3_G-NH_2_ self-assemblies from different PA concentrations: [PA_A_] = 20 mg·mL^−1^; [PA_B_] = 5 mg·mL^−1^; [PA_C_] = 0.05 mg·mL^−1^.

PA self-assemble into fibrils as shown in AFM (Figure 2) with a size and global shape depending on the initial PA concentration going from 0.05 to 20 mg.mL^−1^. Solutions were spin-coated onto a silicon wafer in sort that a water evaporation along with solution spreading lead to a progressive—although quick—PA concentration driving the PA assembly from almost individualized PA cylindrical micelles (Figure 2C) at low concentration (0.05 mg.mL^−1^) towards larger aggregates, such as ribbon-like fibrils at higher concentration (20 mg.mL^−1^) (Figure 2A). 

**Figure 3:**
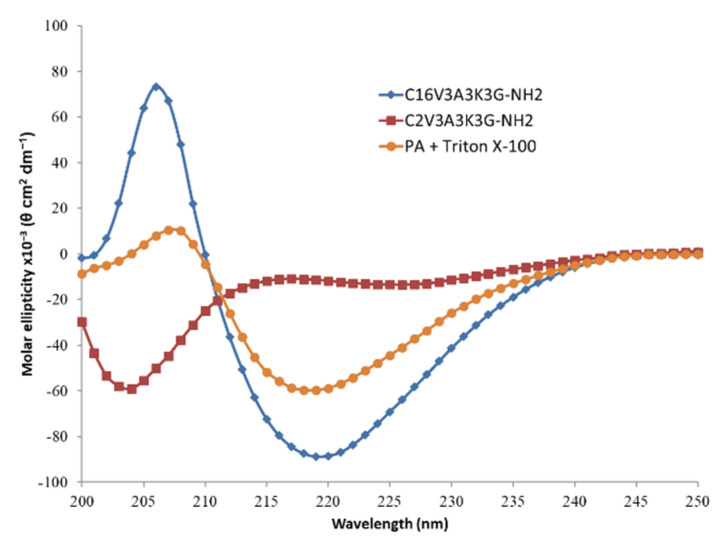
Circular dichroism (CD) spectra of C_16_V_3_A_3_K_3_G-NH_2_ (blue; [C] = 4 g·L^−1^), C_2_V_3_A_3_K_3_G-NH_2_ (red; [C] = 4 g·L^−1^) and C_16_V_3_A_3_K_3_G-NH_2_ ([C] = 4 g·L^−1^) + Triton X-100 ([C] = 1 g·L^−1^) (orange) in H_2_O at 25 °C.

The signature of β-sheet formation of the C_16_V_3_A_3_K_3_G-NH_2_ in aqueous solution is confirmed in Circular dichroism (CD) spectroscopy (Figure 3). In addition, the importance of the length of the alkyl chain is also underlined as shown by the signal, shifted to random coil, observed with the same PA except with regard to its hydrophobic part, reduced from a C16 to a C2. PA cylindrical micelles could be disturbed by the presence of other amphiphilic molecules, such as surfactant. As an illustration, CD spectrum of the PA in presence of Triton X-100 surfactant shows a decrease in CD intensity at 206 nm which is a marker of β-sheet content.

PA cylinders were then used as structuring agent for the membrane formation and electrostatic interactions with PSSNa were promoted to yield membranes able to sustain water pressure (Figure 1). PSSNa is a polyanion able to interact with the K_3_ sequence of the lipidated PA as demonstrated in literature [24]. In doing so, PSSNa should be able to amplify the cylindrical structure of the PA micelle to another scale allowing the formation of gels or functional materials. Concretely, the polymer will line the external surface of the PA micelles via electrostatic interactions. Therefore, the balance between the number of charges brought by the PA and the number of charges brought by the polymer is an important parameter.

**Figure 4:**
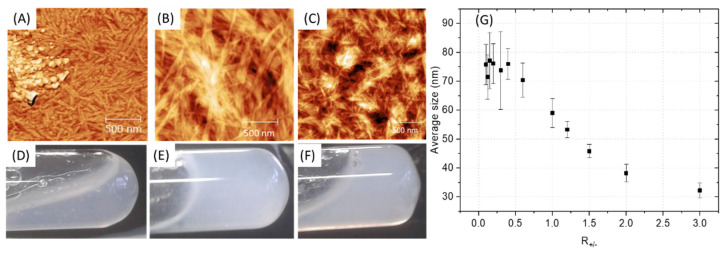
(**A**–**C**) AFM of PA-PSSNa mixtures at different ratios of PA (+) charges/polymer (−) charges (R_+/−_). (**D**–**F**) Digital photographs of the PA-polymer gels. (**A**,**D**): R_+/−_ = 1/3. (**B**,**E**): R_+/−_ = 1. (**C**,**F**): R_+/−_ = 3. (**G**) Average fibril diameter, estimated by AFM, as a function of the PA (+) charges /polymer (−) charge ratios (R_+/−_), standard deviation.

Depending on the ratio PA (+) charges /polymer (−) charges (R_+/−_) used, the appearance of a gel was visually observed with the highest rigidity when R_+/−_ is equal to 1, that is to say for a total compensation of electrostatic charges (Figure 4D–F). However, it is advisable to be very careful with this indicator, which should only be considered as a relative reference. Indeed, R_+/−_ assumes that all charges are accessible which cannot be the case with the use of polymer due to the steric hindrance and chain folding. In addition, when the two aqueous solutions are put into contact, an almost instantaneous structuring of the interface occurs, then limiting the inter-diffusion of the solutions [17]. Therefore, the charge balance is in fact distorted by a partial segregation of the solutions components. AFM images show a similar material organization for R_+/−_ > 1 with a polymer homogeneously distributed within the nanostructure (Figure 4C). However, in the presence of an excess of polymer (R_+/−_ < 1), inhomogeneity can be observed with the appearance of accumulation zones of the polymer which no longer integrate the PA network. The change in the average fibril diameter as a function of R_+/−_ (Figure 4G) shows a plateau regime in the presence of a polymer excess followed by a size decrease while increasing the PA amount in the mixture. It seems logical to observe an increase in the size of the fibrils when increasing the polymer concentration if we consider that the polymer coils between the PA micelles and aggregates them. However, beyond a critical threshold (R_+/−_~0.6), which differs from the theoretical value as explained previously, the increase in the size of the fibrils stops, the polymer is no longer integrated into the PA network and accumulates. The excess of polymer is undesirable because it induces unstructured areas formed from water-soluble polymers that may gradually dissolve and degrade the final material. To minimize these heterogeneity problems in the material, a R_+/−_ equal to 1 was targeted for the rest of this work, leading to formation of fibrils with an approximate size of 60 nm.

**Figure 5:**
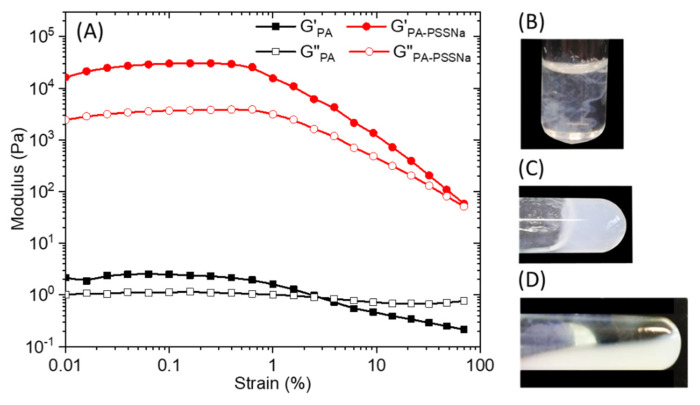
(**A**) Storage (G’) and loss (G’’) modulus of a 10 wt.% solution of C_16_V_3_A_3_K_3_G-NH_2_ (black) or C_16_V_3_A_3_K_3_G-NH_2_—PSSNa (red) in water as a function of strain at a constant frequency of 10 rad/s. Aqueous solution mixture between C_16_-V_3_A_3_K_3_G-NH_2_ (15 mg in 0.5 mL mQ water) and PSSNa (6 mg in 0.5 mL mQ water) (R_+/−_ = 1) just after complete addition (**B**), after slight hand stirring (**C**), and after vortexing (**D**).

Strain sweep rheological experiments performed on 10 wt.% C_16_V_3_A_3_K_3_G-NH_2_ aqueous solution (Figure 5A) at a constant frequency of 10 rad/s, showed that the mechanical properties deviated from the linear viscoelastic region above 0.5 % strain. Overall moduli are low when the PA is alone and the cross-over point from gel to liquid (G′ = G″) occurred at about 3% strain. When the aqueous solutions of PA and polymer, prepared separately, are brought into contact, the medium becomes cloudy instantly (Figure 5B). A simple hand stirring makes it possible to homogenize the medium which forms a gel (Figure 5C). However, this gel is relatively weak and can break down irreversibly upon vortexing (Figure 5D). The presence of PSSNa increases the storage and the loss moduli by 4 order of magnitude and the cross-over point was pushed towards values of strain close to 100%. 

**Figure 6:**
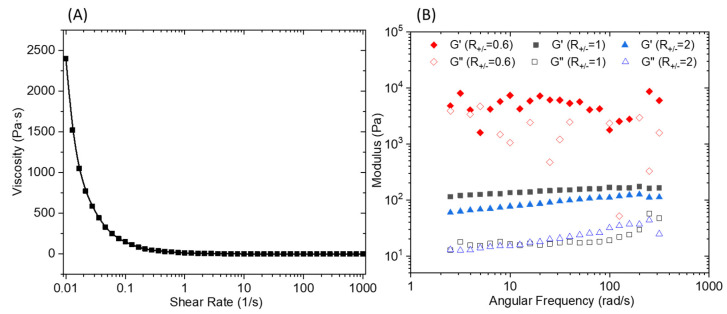
(**A**) Viscosity versus shear rate of the PA/PSSNa physical gel. (**B**) Storage (G’) and loss (G’’) modulus for 10 wt.% solutions of C_16_V_3_A_3_K_3_G-NH_2_–PSSNa in water as a function of frequency at a constant strain of 0.1% and different R_+/−_.

An oscillatory frequency sweep performed (Figure 6B) at a constant strain of 0.1% shows an almost independent frequency behavior over the explored range. It should be noted that for an R_+/−_ of 0.6, the curve presents an erratic shape which is the result of a macroscopic separation observed under shear. The flow curve presented in Figure 6A demonstrates a marked shear thinning behavior. The elaboration of a membrane material from C_16_V_3_A_3_K_3_G-NH_2_–PSSNa mixtures requires casting a solution that has a sufficient viscosity to control the final material thickness and thus avoid the presence of imperfection linked to a film that is too thin. Consequently, a premixing of C_16_V_3_A_3_K_3_G-NH_2_ and PSSNa solutions followed by casting on a support will not make it possible to obtain the expected morphology due to the too low viscosity of the solution under shear. One strategy then consists of depositing the two aqueous solutions successively and allowing the gel to form directly under its final geometry without subsequent manipulation.

**Figure 7:**
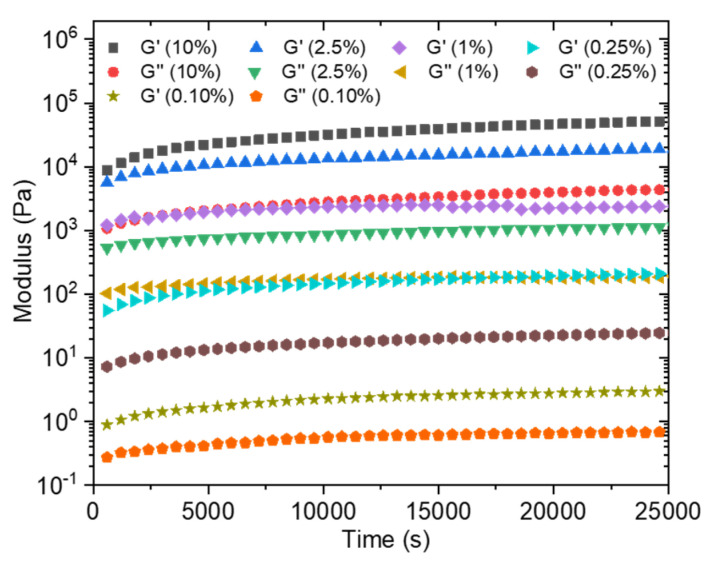
Storage (G’) and loss (G’’) modulus of C_16_V_3_A_3_K_3_G-NH_2_–PSSNa in water as a function of time at different solid concentrations (frequency = 10 rad/s; strain = 0.1%; R_+/−_ = 1).

**Figure 8:**
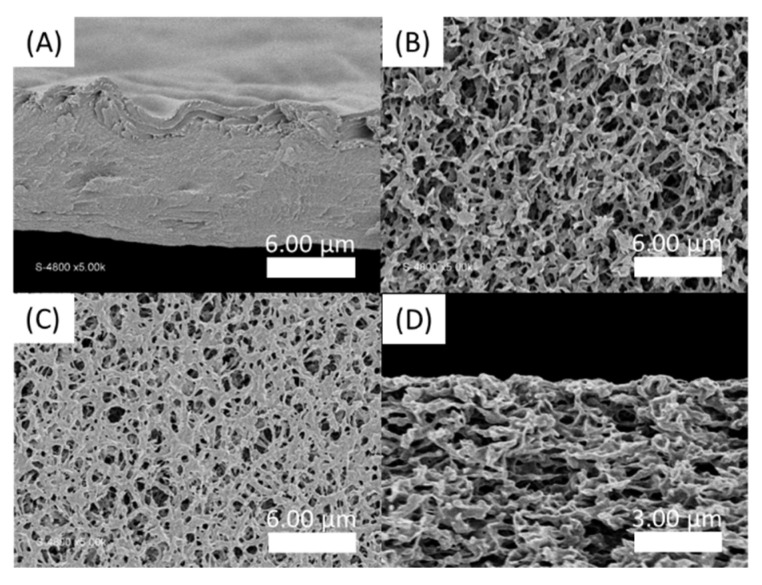
SEM pictures of PA-PSSNa films. (**A**) Cross section of the self-standing membrane formed by tape-casting of a PSSNa solution then covered with C_16_V_3_A_3_K_3_G-NH_2_ solution; (**B**) Top view of virgin hydrophilic PVDF membrane; (**C**) Top view and (**D**) Cross section SEM images of the film deposited on hydrophilic PVDF by impregnation of PSSNa followed by C_16_V_3_A_3_K_3_G-NH_2_ tape-casting.

In order to estimate the time required for the formation of a sufficiently solid gel before immersing the obtained membrane in a water bath to wash out the unreacted precursors, the loss and storage moduli were followed over time at different concentrations going from 0.1 wt% to 10 wt% (Figure 7). Moduli values logically increase with the total solid concentration. The kinetic monitoring shows two stages with a first part characterized by a steep increase in moduli following the contacting of the aqueous solutions. It has to be noted that the time, although short, necessary to start the analysis after mixing the solutions does not allow us to observe the initiation of this step. On the other hand, this rapid increase is followed by a quasi-plateau for which the moduli evolve only modestly. This observation is in agreement with the previously cited literature on the rapidity of formation of a solid interface in the case of a strong interaction between the PA and the polymer, which is precisely our case. Conventionally, the membranes are produced by a phase inversion process in which the polymer in a good solvent is immersed in a non-solvent which allows the appearance of a phase rich in polymer (matrix) and a phase poor in polymer (pores). This technique cannot be carried out here because of the high attraction between the two components, which is moreover a conventional problem with polyelectrolyte layers. In a first attempt, a PSSNa solution was tape-casted onto a glass support, then completely covered with a solution of the PA. After a waiting time of 2 h 30 min, the support is then immersed in a water bath so as to wash off the excess unreacted products. A self-supporting material of about 9 µm in thickness (as judged by SEM images of the film cross sections) is obtained (Figure 8A) but it was too brittle to withstand water pressure during filtration. 

Thus, in a second attempt, a supported membrane with a PVDF ultrafiltration membrane as a support layer was developed. For this, the PVDF membrane is first immersed in a solution of PSSNa. The C_16_V_3_A_3_K_3_G-NH_2_ solution is then tape-casted on the surface of the impregnated support. After a waiting time of 2h30, the film is immersed in demineralized water. Figure 8C shows an apparent decrease in the surface porosity of the PVDF membrane, compared to the virgin membrane (Figure 8B). However, the coverage is only partial and the presence of this selective layer in the thickness of the material is not observed (Figure 8D).

**Figure 9:**
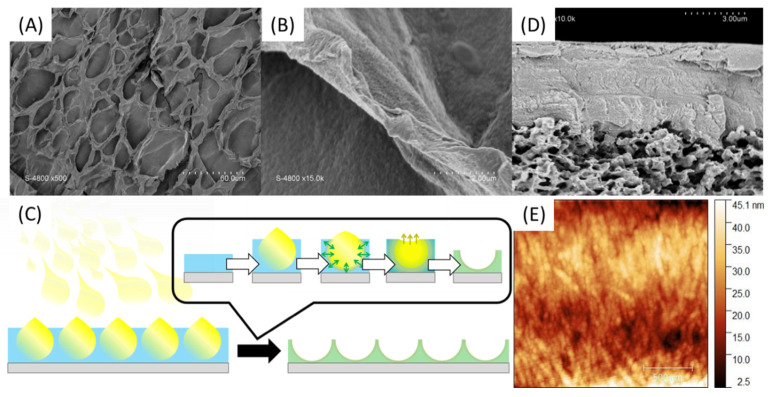
SEM surface pictures of PA-PSSNa self-standing membranes made by tape-casting of a PA solution followed by spray-coating of PSSNa solution (**A**,**B**) as illustrated in (**C**). SEM pictures of cross section (**D**) and AFM (**E**) of the PVDF-supported membrane.

In order to enhance the chance of forming a continuous membrane selective layer rather than randomly distributed inside the PVDF commercial membrane material, spray-coating technique was explored. In a first attempt, a solution of PA was tape-casted on a glass support and then a solution of PSSNa was sprayed on the surface so as to form fine droplets which will coalesce to form a thin layer. As shown in Figure 9A,B, a material is well formed but has a surface punctuated with cavities. These are the result of a rapid interaction between the PSSNa and the PA since the interface seems to have been formed when the PSSNa droplets came in touch with the aqueous layer of PA (Figure 9C), without allowing the time required for these droplets to coalesce in a continuous layer. Although the result is interesting by illustrating how fast are the interactions to yield a solid interface, the material obtained is too fragile for the envisaged application. Finally, when a PSSNa solution was tape-casted on a PVDF support membrane, followed by a spray of the PA solution, a relatively uniform material is obtained (Figure 9D). The imprint left by the droplets is much less present as shown by the cross section of the material. The thickness of the selective layer is 4.5 µm. Good adhesion is observed between the PVDF support and the self-assembled PA-PSSNa selective layer. The mechanical properties are also better and allowed an AFM analysis which demonstrates the presence of structuring network of fibers as expected (Figure 9E). The diameter of the fibers are of the order of 45 nm. 

**Figure 10:**
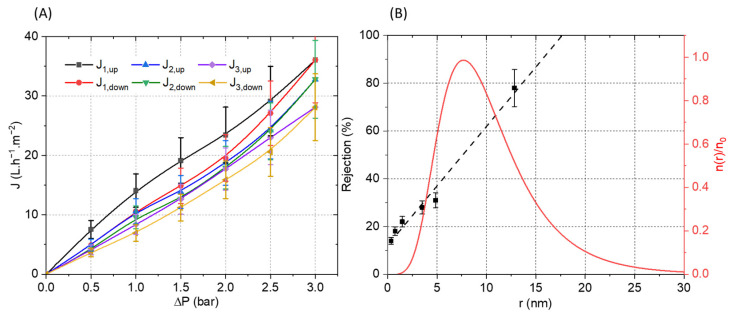
(**A**) Pressure increase and decrease cycles of water permeation experiment with the PVDF-supported membrane. (**B**) Polyethylene glycol (PEG) rejection coefficient (R) vs. the Einstein–Stokes radius of the solute (PEG) (black). Log-normal pore size distribution: reduced number of pores per unit of area n(r)/n_0_ vs. the pore radius (red).

The membrane was then mounted in a dead-end filtration cell and three full pressure cycles (increasing and decreasing) were performed (Figure 10). Relatively linear curves with a low hysteresis between the up and down cycles were observed. The calculated membrane permeability of 10 L·h^−1^·m^−2^·bar^−1^ is moderate, which could be ascribed to the relatively high thickness of the selective layer (4.5 µm). The slight decrease in flux observed between the filtration cycles can be explained by the electrostatic nature of the interactions between the PA and PSSNa. Indeed, it is possible that a reorganization of the interactions under pressure could be at the origin of this moderate decrease by compressing the selective layer. The pore size of the membrane was estimated from filtration experiments using polyethylene glycol (PEG) of different molecular weights. The solute rejection versus the solute diameter is plotted in Figure 10B. Using the Einstein–Stokes radius of the solute, the mean pore size is considered as the solute diameter that corresponds to a retention of 50%, whereas the geometric standard deviation is obtained from the ratio of solute diameter at retentions of 84.13% and 50% [34]. The membrane material falls into the ultrafiltration range with a pore radius of 7.61 ± 1.90 nm. Based on these values, the pore size distribution was plotted in Figure 10B by using a log-normal model [35].To the best of our knowledge, this is the first example of a composite filtration membrane formed from water-soluble polyelectrolytes structured by a self-assembling peptide. 

## 4. Conclusions

In conclusion, we have reported a novel method to prepare nanofibrous network able to sustain the water pressure in the scope of membrane filtration. Nanocylinders of C_16_-V_3_A_3_K_3_G-NH_2_ were synthesized and their co-assembly with PSSNa chains were explored to produce the targeted materials. The charge ratio between the peptide amphiphiles and the polymer was optimized to link to nanocylinders together via electrostatic interactions. The tape-casting of PSSNa solution on a PVDF support membrane, followed by a spray-coating of the C_16_-V_3_A_3_K_3_G-NH_2_ solution produced a relatively uniform membrane with a thickness of 4.5 µm able to sustain a water pressure of 3 bars.

## Data Availability

If you need to have access to some of the raw data, please contact the corresponding author.
